# The effect of an oral nutritional supplement enriched with fish oil on weight-loss in patients with pancreatic cancer

**DOI:** 10.1038/sj.bjc.6690654

**Published:** 1999-09

**Authors:** M D Barber, J A Ross, A C Voss, M J Tisdale, K C H Fearon

**Affiliations:** University Department of Surgery, Royal Infirmary of Edinburgh, Edinburgh , EH8 9JA, UK; Ross Products Division, Abbot Laboratories, Columbus, 43215-1724, USA; Department of Pharmaceutical Sciences, Aston University, Birmingham, B4 7ET, UK

**Keywords:** pancreatic cancer, cachexia, eicosapentaenoic acid, docosahexaenoic acid, fish oil, nutritional supplementation

## Abstract

Previous studies have suggested that administration of oral eicosapentaenoic acid (EPA) will stabilize weight in patients with advanced pancreatic cancer. The aim of the present study was to determine if a combination of EPA with a conventional oral nutritional supplement could produce weight gain in these patients. Twenty patients with unresectable pancreatic adenocarcinoma were asked to consume two cans of a fish oil-enriched nutritional supplement per day in addition to their normal food intake. Each can contained 310 kcal, 16.1 g protein and 1.09 g EPA. Patients were assessed for weight, body composition, dietary intake, resting energy expenditure (REE) and performance status. Patients consumed a median of 1.9 cans day^−1^. All patients were losing weight at baseline at a median rate of 2.9 kg month^−1^. After administration of the fish oil-enriched supplement, patients had significant weight-gain at both 3 (median 1 kg, *P* = 0.024) and 7 weeks (median 2 kg, *P* = 0.033). Dietary intake increased significantly by almost 400 kcal day^−1^ (*P* = 0.002). REE per kg body weight and per kg lean body mass fell significantly. Performance status and appetite were significantly improved at 3 weeks. In contrast to previous studies of oral conventional nutritional supplements in weight-losing cancer patients, this study suggests that an EPA-enriched supplement may reverse cachexia in advanced pancreatic cancer. © 1999 Cancer Research Campaign


*British Journal of Cancer* (1999) **81**(1), 80–86
© 1999 Cancer Research Campaign
Article no. bjoc.1999.0654

### The effect of an oral nutritional supplement enriched with fish oil on weight-loss in patients with pancreatic cancer

**MD Barber[1], JA Ross[1], AC Voss[2], MJ Tisdale[3] and KCH Fearon[1]**

[1]University Department of Surgery, Royal Infirmary of Edinburgh, Edinburgh EH8 9JA, UK; [2]Ross Products Division, Abbot Laboratories, 625 Cleveland Avenue, Columbus, Ohio 43215-1724, USA; [3]Department of Pharmaceutical Sciences, Aston University, Birmingham B4 7ET, UK

**Summary** Previous studies have suggested that administration of oral eicosapentaenoic acid (EPA) will stabilize weight in patients with advanced pancreatic cancer. The aim of the present study was to determine if a combination of EPA with a conventional oral nutritional supplement could produce weight gain in these patients. Twenty patients with unresectable pancreatic adenocarcinoma were asked to consume two cans of a fish oil-enriched nutritional supplement per day in addition to their normal food intake. Each can contained 310 kcal, 16.1 g protein and 1.09 g EPA. Patients were assessed for weight, body composition, dietary intake, resting energy expenditure (REE) and performance status. Patients consumed a median of 1.9 cans day$^{-1}$. All patients were losing weight at baseline at a median rate of 2.9 kg month$^{-1}$. After administration of the fish oil-enriched supplement, patients had significant weight-gain at both 3 (median 1 kg, $P = 0.024$) and 7 weeks (median 2 kg, $P = 0.033$). Dietary intake increased significantly by almost 400 kcal day$^{-1}$ ($P = 0.002$). REE per kg body weight and per kg lean body mass fell significantly. Performance status and appetite were significantly improved at 3 weeks. In contrast to previous studies of oral conventional nutritional supplements in weight-losing cancer patients, this study suggests that an EPA-enriched supplement may reverse cachexia in advanced pancreatic cancer.

**Keywords:** pancreatic cancer; cachexia; eicosapentaenoic acid; docosahexaenoic acid; fish oil; nutritional supplementation

Pancreatic cancer is almost inevitably associated with progressive nutritional decline (Wigmore et al, 1997*a*). Weight-loss in patients with gastrointestinal cancer is often refractory to therapeutic intervention and is associated with a shorter survival time and a reduced quality of life (DeWys et al, 1980; Ovesen et al, 1993*a*). The provision of conventional oral nutritional supplements may increase overall dietary intake but this does not generally lead to any benefit in terms of nutritional status (Evans et al, 1987; Ovesen et al, 1993*b*). Consequently it has been suggested that the metabolic processes which contribute to weight-loss in patients with cancer may also block the accretion of lean tissue (Moldawer and Copeland, 1997).

Pro-inflammatory cytokines, notably interleukin 6 (IL-6), can induce a cachectic state when injected into animals and monoclonal antibodies to such cytokines may attenuate certain features of cachexia in tumour-bearing animals (McNamara et al, 1992). The acute phase protein response (APPR) has been shown to be associated with increased resting energy expenditure (Falconer et al, 1994*a*) and to correlate with reduced nutritional intake (Wigmore et al, 1997*b*) in weight-losing patients with pancreatic cancer. In addition, the APPR has been demonstrated to be the strongest independent predictor of poor prognosis in patients with pancreatic cancer (Falconer et al, 1995). The APPR is modulated primarily by pro-inflammatory cytokines, including IL-6 (Heinrich et al, 1990). Thus there appears to be a strong link between pro-inflammatory cytokine activity and the development of cachexia in patients with pancreatic cancer. Attempts to manipulate the inflammatory response in cancer patients with the intention of improving nutritional status have been made previously with promising results (McMillan et al, 1997).

The n-3 polyunsaturated fatty acids, eicosapentaenoic acid (EPA) and docosahexaenoic acid (DHA), are immunomodulatory and have been shown to suppress endotoxin-induced production of pro-inflammatory cytokines such as IL-1 and tumour necrosis factor (TNF) by peripheral blood mononuclear cells (PBMC) from healthy volunteers (Meydani et al, 1993). Studies of weight-losing pancreatic cancer patients receiving high-purity EPA have demonstrated suppression of PBMC IL-6 production (Wigmore et al, 1997*c*). EPA has also been shown to have inhibitory effects on the growth of human pancreatic cancer cell lines in vitro (Falconer et al, 1994*b*) and to have anti-tumour and anti-cachectic effects in the chemoresistant murine MAC16 colon adenocarcinoma model (Beck et al, 1991). The cachexia seen in this animal model has been attributed to the production of a proteolysis inducing factor by tumour cells and such a factor is also found in tumour-bearing humans with cachexia (Todorov et al, 1996). It has been suggested that EPA may act by inhibiting the end organ effects of this factor (Tisdale, 1996).

We have previously reported that administration of a mixed fish oil preparation (providing around 2.2 g EPA and 1.4 g DHA daily) and a pure EPA preparation (providing 6 g EPA daily) will stabilize weight in patients with unresectable pancreatic cancer (Wigmore et al, 1996; Barber et al, 1997). Clearly, in order to lay down new tissue and thereby increase body weight additional macronutrients need to be consumed.

*Received 3 September 1998*
*Revised 11 February 1999*
*Accepted 12 April 1999*

*Correspondence to:* KCH Fearon

The present study aimed to assess whether the provision of additional oral calories and protein in conjunction with fish oil could reverse weight-loss in patients with advanced pancreatic cancer.

### MATERIALS AND METHODS

### Patients

Patients enrolled were men or non-pregnant, non-lactating women between 18 and 80 years with histological confirmation or unequivocal operative or radiological diagnosis of unresectable adenocarcinoma of the pancreas with evidence of ongoing weight-loss. Patients had a life expectancy of over 2 months and a WHO performance status ≤ 2 at enrolment. Written, informed consent was obtained from all patients. Patients were excluded if they had received surgery or endoscopic stenting during the previous 4 weeks, had other active medical conditions, another malignancy or were receiving medication which could profoundly modulate metabolism or weight. No patients had received radiotherapy or chemotherapy. The study protocol was approved by the Lothian Ethical Committee. A total of 20 patients were recruited to the study. Confirmation of unresectable pancreatic adenocarcinoma was by histology (16/20) or unequivocal operative or radiological findings (4/20). Seventeen patients had cancers of the head of pancreas and three of the body. On enrolment, none of the patients were jaundiced, pyrexial, ascitic, severely anaemic or had clinical or radiological evidence of infection and none were taking steroid drugs. All patients had adequate pain control at the time of study. Pancreatic enzyme supplements were administered if patients had or developed clinical evidence of steatorrhoea.

Patients were studied formally at baseline and 3 and 7 weeks, although patients were assessed approximately monthly until death, withdrawal from the study or until their condition deteriorated such that further assessment was not possible. Trial results were monitored independently.

### Fish oil-enriched nutritional supplement

The fish oil-enriched nutritional supplement was provided by Ross Products Division, Abbott Laboratories, Columbus, Ohio, USA. The composition of this product is shown in Appendix 1. Patients were requested to store product in the refrigerator.

Patients were asked to consume two cans per day (providing 610 kcal, 32.2 g protein, 2.2 g EPA and 0.96 g DHA). Compliance was assessed by a diary of consumption, return of labels from empty cans and by plasma fatty acid analysis at baseline and after 3 weeks' consumption of the supplement.

### Toxicity assessment

Blood was drawn for full blood count, electrolytes, urea, glucose and liver function tests. These were measured using standard automated techniques in the haematology and clinical chemistry laboratories of the Royal Infirmary, Edinburgh, UK. A history and full clinical examination was performed at each of these visits. Toxicity reported to general practitioners or observed on clinical examination was documented.

### Nutritional and metabolic assessment

#### *Anthropometry*

At the initial assessment, height, pre-illness stable weight and duration of weight-loss were recorded. Subjects were weighed on spring balance scales (Seca, Germany) without shoes and wearing light clothing. Mid-arm muscle circumference (MAMC) was calculated using Jelliffe's equation (Jelliffe, 1966). Triceps skinfold thickness (TSF) was measured using Harpenden calipers (John Bull British Indicators Ltd, UK). Three measurements were performed and the mean value recorded.

#### *Body composition analysis*

Body composition was measured using a Xitron 4000B multiple frequency bio-electrical impedence analyser (Xitron Technologies, San Diego, CA, USA). All assessments were made with the subject supine and limbs apart. Electrodes were placed over the wrist and ankle joints and metacarpal and metatarsal heads. Repeat measurements were performed using the same pair of limbs. Resistance was measured at 200 Hz. Values for total body water were derived using equations validated in a similar patient group (Hannan et al, 1995). Lean body mass was calculated assuming that lean tissue contains 73% water (Shizgal, 1990).

#### *Nutritional intake*

The daily caloric intake of patients was measured at baseline and after 3 weeks of supplement administration. Values were based on the mean intake recorded in a 3-day food diary calculated using CompEat 4 software (Nutrition Systems, London, UK).

#### *Energy expenditure*

At baseline and after 3 weeks resting energy expenditure (REE) was measured as described previously (Falconer et al, 1994*a*) after an overnight fast using a ventilated hood system (Deltatrac II, Datex, Helsinki, Finland). Patients attended at 08:00 h and rested for at least 30 min in the supine position. Measurements were made over at least 30 min with patients supine in a thermoneutral environment. Measurements for the initial 10 min were discarded and the remaining values averaged. The equipment was calibrated before each measurement.

#### *Acute phase protein response*

The APPR was documented by assaying serum C-reactive protein (CRP) (Abbott Diagnostic, Maidenhead, UK). The limit of detection of this assay is 10 mg $l^{-1}$.

#### *Fatty acid analysis*

Plasma phospholipid fatty acid analysis was performed by gas chromatography as described previously (Wigmore et al, 1996) before commencement of the supplement and 3 weeks thereafter.

### Performance status and appetite

Karnofsky performance status was noted before commencement of the supplement, at three weeks and at monthly intervals thereafter. Appetite was measured on a numerical rating scale between 0 and 10, where 0 indicated absolutely no appetite and 10 indicated an extremely good appetite (Simons et al, 1996).

### Survival

Survival was recorded from the time of diagnosis and study baseline to the time of death. Diagnosis was defined as the date of confirmation of adenocarcinoma of the pancreas by histological examination or of unequivocal operative or radiological findings in patients in whom definitive pathological confirmation of diagnosis was lacking.

© 1999 Cancer Research Campaign 

**Table 1** Baseline characteristics of study patients with advanced pancreatic cancer ($n = 20$)

| | |
|---|---|
| Weight (kg) | 55.2 (48.8–61.2) |
| Body mass index (kg m$^{-2}$) | 19.8 (17.8–21.8) |
| Rate of weight change per month (kg) | –2.9 (–4.4—2.2) |
| Percentage weight loss | 17.9 (15.9–22.8) |
| Mid arm muscle circumference (cm) | 20.8 (18.4–22.4) |
| Triceps skinfold thickness (mm) | 11.1 (7.8–15.9) |
| Percentage total body water | 52.9 (51.2–56.1) |
| Lean body mass (kg) | 41.5 (38.1–44.8) |
| Fat mass (kg) | 14.5 (12.3–16.7) |
| C-reactive protein (mg l$^{-1}$) | 10 (<10–27) |
| Karnofsky performance status[a] | 85 (80–90) |
| Appetite[b] | 5 (3–7) |

Figures are median (interquartile range). [a]From a score of 10 (moribund) to 100 (normal). [b]From a score of 0 (none) to 10 (excellent).

**Table 2** Change in characteristics of patients with advanced pancreatic cancer after 3 and 7 weeks' administration of a fish oil-enriched nutritional supplement.

| Duration of supplement administration | 3 weeks | | 7 weeks | |
|---|---|---|---|---|
| Number of patients | 18 | | 13 | |
| | | *P* | | *P* |
| Weight change (kg) | +1.0 (–0.1 ± 2.0) | 0.024 | +2.0 (–0.4 ± 4.6) | 0.033 |
| Change in mid arm muscle circumference (cm) | 0 (–0.2 ± 0.5) | NS | +0.4 (–0.7 ± 1.1) | NS |
| Change in triceps skinfold thickness (mm) | 0 (–0.2 ± 0.4) | NS | +0.2 (–0.5 ± 0.6) | NS |
| Change in percentage total body water | +0.1 (–0.3 ± 1.9) | NS | –0.1 (–0.4 ± 1.5) | NS |
| Change in lean body mass (kg) | +1.0 (+0.6 ± 1.4) | 0.0064 | +1.9 (+1.0 ± 3.0) | 0.0047 |
| Change in fat mass (kg) | –0.2 (–0.8 ± 0.9) | NS | +0.2 (–0.8 ± 2) | NS |
| Change in C-reactive protein (mg/l) | 0 (0 ± 3) | NS | 0 (–5 ± 22) | NS |
| Change in Karnofsky performance status[a] | +10 (0 ± 10) | 0.0047 | +10 (0 ± 10) | 0.046 |
| Change in appetite[b] | +1 (0 ± 1) | 0.001 | +1 (0 ± 1) | NS |

Figures are median (interquartile range). [a]From a score of 10 (moribund) to 100 (normal). [b]From a score of 0 (none) to 10 (excellent). Paired comparison with baseline by Wilcoxon signed rank test.

### Statistical analysis

Data are presented as the median and interquartile range. The study was analysed on an intention-to-treat basis. Due to the inevitable deterioration of patients in such a study of a palliative intervention, of 20 patients initially enrolled, data were available on 18 patients at 3 weeks and 13 patients at 7 weeks. Paired comparisons with baseline values were performed using Wilcoxon signed rank test. A *P*-value of less than 0.05 was taken to denote significance.

### RESULTS

### Patient characteristics

Patients comprised ten males and ten females of median age 62 years (range 51–75). Tumour stages were as follows: stage 2, eight patients; stage 3, three patients; and stage 4, nine patients. No patients underwent prior chemotherapy or radiotherapy. Eight patients underwent non-resectional palliative surgical procedures and eight patients had endobiliary stenting prior to the study. Patients had tumours of the pancreatic head in 17 cases and the pancreatic body in the remainder. Baseline characteristics of patients is shown in Table 1. Of 20 patients enrolled, 18 were available for analysis at week 3 and 13 patients at week 7. One patient on whom data was available at 7 weeks had discontinued the supplement at week 3 due to excessive weight gain. No patients on whom data was available over the 7-week study period developed clinically detectable ascites or oedema.

### Tolerance/toxicity

Patients consumed a median of 1.9 cans of the fish oil-enriched supplement per day (range 1.2–2). Full blood count, electrolytes, urea, glucose and liver function tests were assessed at each review. Values did not change significantly over the course of the study (data not shown).

Two patients developed and one patient had worsening of pre-existing steatorrhoea with administration of the supplement. This was successfully treated by pancreatic enzyme supplementation. Otherwise no patients developed adverse events which would not be expected with the normal progression of advanced pancreatic cancer.

At 3 weeks two patients were unavailable for analysis due to disease progression. At 7 weeks a further five patients were unavailable due to disease progression. Reasons for stopping the supplement during the 7-week study period were temporary interruption in supply in one patient (after 3 weeks), excessive weight gain in one patient (after 3 weeks), dislike of taste in two patients (after 3 weeks) and progression of disease in the remainder. Data were only available for one patient not taking the supplement at 7 weeks. Thus all 18 patients assessed at 3 weeks and 12 of 13 patients assessed at 7 weeks were consuming the supplement.

### Body weight

Changes in body weight for individual patients are depicted graphically in Figure 1. Weight change at 3 ($n = 18$) and 7 ($n = 13$) weeks is shown in Table 2. Patients had statistically significant weight gain at 3 and 7 weeks of median 1 and 2 kg respectively. The patient who had discontinued the supplement at 3 weeks but on whom data was available at 7 weeks gained 0.5 kg over the first 3 weeks on the supplement and lost 0.9 kg in the subsequent 4 weeks off the supplement. Not including this patient, median weight gain at 7 weeks was 2.5 kg (0.2–4.6).

 © 1999 Cancer Research Campaign

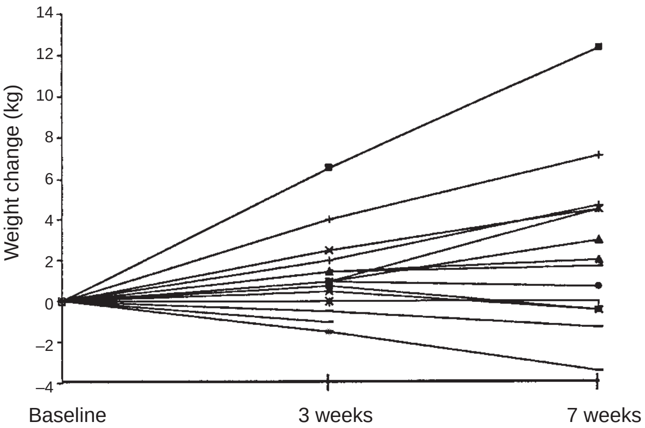

**Figure 1** Weight change of 20 patients with advanced pancreatic cancer administered a fish oil-enriched nutritional supplement after 3 and 7 weeks. Median rate of weight loss prior to intervention was 2.9 kg per month. Median weight gain at 3 weeks was 1.0 kg ($P$=0.028 vs baseline Wilcoxon signed rank test). Median weight gain at 7 weeks was 2.0 kg ($P$=0.033)

### Anthropometry and body composition analysis

Values for MAMC and TSF recorded before commencement of the supplement and changes after 3 and 7 weeks are shown in Tables 1 and 2. There was no significant change in either MAMC or TSF over this period.

Values for total body water expressed as a percentage of total body weight are also presented in Tables 1 and 2. There was no significant change in this factor in patients remaining in the trial at 3 and 7 weeks.

Values for calculated lean body mass and fat mass are presented in Tables 1 and 2. There was a statistically significant rise in calculated lean body mass at 3 and 7 weeks (of median 1.0 kg and 1.9 kg respectively) but no change in fat mass at either time point.

### Nutritional intake

The daily caloric intake of patients before commencement of supplement and after 3 weeks ($n$ = 18) are presented in Table 3. There was a statistically significant increase in caloric intake after 3 weeks ($P$ = 0.0016). The median increase for each patient was 372 kcal day$^{-1}$.

### Energy expenditure

REE and REE expressed per kg body weight and per kg lean body mass as measured at baseline and after 3 weeks are presented in Table 3. While there was no change in overall REE ($P$ = 0.18), REE kg body weight$^{-1}$ and REE kg lean body mass$^{-1}$ both fell significantly ($P$ = 0.025 and 0.018 respectively).

### Acute phase protein response

Values for serum CRP concentration before commencement of the supplement and changes after 3 and 7 weeks are shown in Tables 1 and 2. There was also no change in the percentage of patients exhibiting an APPR as defined by a CRP ≥ 10 mg l$^{-1}$ throughout the study period.

### Fatty acid analysis

The EPA, DHA and arachidonic acid content of plasma phospholipids of patients before commencement of supplement and after 3 weeks are presented in Table 4. There was a statistically significant increase in both EPA and DHA in plasma phospholipids after 3 weeks.

### Performance status and appetite

Scores for Karnofsky performance status before administration of the supplement and change after 3 and 7 weeks are shown in Tables 1 and 2. There was a statistically significant improvement in Karnofsky performance status compared with baseline after 3 and 7 weeks administration of the supplement ($P$ = 0.0047 and 0.046 respectively).

Appetite scores before administration of the supplement and change after 3 and 7 weeks are shown in Tables 1 and 2. There was a statistically significant improvement in appetite at 3 weeks ($P$ = 0.0095).

### Survival

Nineteen of the patients have died. One patient remains alive at 14 months from the time of diagnosis. The survivor has histological confirmation of the diagnosis of pancreatic adenocarcinoma. The censored median survival from the time of diagnosis for all patients was 254 days (124–311). The censored median survival from the time of commencing the supplement was 170 days (90–270).

**Table 3** Caloric intake and resting energy expenditure of 18 patients with advanced pancreatic cancer at baseline and after 3 weeks' administration of a fish oil-enriched nutritional supplement

| Duration of supplement administration | Caloric intake (kcal day$^{-1}$) | REE (kcal day$^{-1}$) | REE/kg body weight (kcal kg$^{-1}$ day$^{-1}$) | REE/kg lean body mass (kcal kg$^{-1}$ day$^{-1}$) |
|---|---|---|---|---|
| 0 | 1450 | 1339 | 24.2 | 34.0 |
| | (1048–2043) | (1159–1448) | (23.1–27.7) | (29.6–35.6) |
| 3 weeks | 1798[a] | 1303[b] | 24.0[c] | 31.8[d] |
| | (1355–2474) | (1186–1470) | (20.2–25.8) | (27.7–33.9) |

Figures are median (interquartile range). REE, resting energy expenditure. Paired comparison of value at 3 weeks versus baseline using Wilcoxon signed rank test: [a]$P$ = 0.0016; [b]$P$ = 0.18; [c]$P$ = 0.025; [d]$P$ = 0.018.

© 1999 Cancer Research Campaign 

**Table 4** Percentage of EPA, DHA and AA in plasma phospholipid profile of 18 patients with advanced pancreatic cancer at baseline and after 3 weeks' administration of a fish oil-enriched nutritional supplement

| Duration of supplement administration | EPA (%) | DHA (%) | AA (%) |
|---|---|---|---|
| 0 | 0.5 (0.2–1.6) | 2.9 (1.6–3.9) | 6.4 (4.1–7.5) |
| 3 | 5.2 a (2.6–6.7) | 4.8 b (3.3–5.3) | 5.1 c (4.3–6.4) |

Figures are median (interquartile range). EPA, eicosapentaenoic acid; DHA, docosahexaenoic acid; AA, arachidonic acid. Paired comparison of value at 3 weeks versus baseline using Wilcoxon signed rank test: [a]$P$ = 0.0003; [b]$P$ = 0.0086; [c]$P$ = 0.33.

### DISCUSSION

In the present study the administration of a nutritional supplement enriched with fish oil resulted in weight gain in a group of patients with advanced pancreatic cancer who had previously been losing weight at a median rate of 2.9 kg month$^{-1}$.

Not all of the patients who entered the present study completed the 7-week study period. This is a reflection of the advanced stage at presentation and subsequent poor survival of patients with pancreatic cancer. By observing weight change in those survivors who remained on study at 3 ($n$ = 18/20) and 7 ($n$ = 13/20) weeks there might be a bias to overestimate the overall efficacy of EPA as an anti-cachectic agent. Nevertheless, the finding of weight gain in the majority of patients at both time points contrasts markedly with our previous study of patients receiving supportive care alone which demonstrated near uniform progressive weight loss in patients with this disease (Wigmore et al, 1997*a*). In addition, previous controlled trials of oral nutritional supplementation in patients with advanced cancer have succeeded in increasing nutritional intake but have not been able to show any change in weight change compared with controls (Evans et al, 1987; Ovesen et al, 1993*b*). There was a variation in response of patients in the present study with some continuing to lose weight. The factors determining response are not clear as there was no relationship between weight change and disease stage, previous surgery or the acute phase response. Such analysis is complicated by the fact that even patients who continued to lose weight experienced a slowing of their rate of weight loss with the administration of the trial supplement.

It has been reported that increased body weight following parenteral and enteral nutritional support in cancer patients is largely due to the accumulation of body water (Cohn et al, 1982). However, in the present study there was no change in hydration as total body water as a percentage of body weight remained stable. Values for lean body mass and fat mass were derived from bioimpedence measurements. Overall there was a small but statistically significant increase in lean body mass with administration of the supplement while fat mass remained stable. The coefficient of variation for the estimate of lean body mass has been estimated to be around 2 kg (Jensen et al, 1997). Most of the changes documented in this study were around this value and therefore the interpretation of data on individual patients is difficult. Nevertheless, the changes for the group as a whole suggest that patients were laying down lean tissue rather than fat to account for weight gain and this may have contributed to the observed rise in Karnofsky performance status. This contrasts with studies of synthetic progestogens such as medroxyprogesterone acetate and megestrol acetate in similar patients in whom weight gain is due to the accumulation of fat (Loprinzi et al, 1993; Simons et al, 1998). Measurements of MAMC and TSF demonstrated no significant change from baseline levels but this is not surprising given the small changes in bioimpedence-derived body composition seen. However, they do provide supporting evidence that protein and fat reserves were at least stabilized with administration of the fish oil-enriched supplement. This is in contrast to the continuing decline in MAMC and TSF seen in similar patients who undergo no specific intervention (Wigmore et al, 1997*a*).

Patients in this study achieved a significant increase in nutritional intake and a relative fall in resting energy expenditure. Patients did not simply replace normal food intake with the supplement provided. Overall, patients had an improvement in energy balance of around 500 kcal per day. This is entirely compatible with the weight gain seen and may have provided additional energy for activities of daily living, reflected in the observed improvements in Karnofsky performance status. Appetite appeared to increase over the early part of the study period and may account for the overall increase in food intake.

The fish oil-enriched nutritional supplement was well tolerated with all patients able to consume over one can (237 ml) per day and 15 of 18 managing over 1.75 cans per day. The only adverse event attributable to the trial preparation was steatorrhoea in two patients. A further patient described worsening of their pre-existing steatorrhoea. Steatorrhoea is common in patients with advanced pancreatic cancer (Perez et al, 1983).

There was a marked rise in levels of EPA and DHA in plasma phospholipids representing 5.2% and 4.8% respectively of fatty acids after 3 weeks of receiving the supplement. This also provided an objective measure of patient compliance. Such a pattern of change is similar to that previously reported following fish oil supplementation (Leaf and Weber, 1988; Wigmore et al, 1996). The percentage of EPA incorporation achieved is similar to that seen after supplementation with crude fish oil capsules in pancreatic cancer patients given a similar final dose equivalent to 2 g EPA day$^{-1}$ after a dose escalation period (Wigmore et al, 1996).

We have previously demonstrated down-regulation of pro-inflammatory cytokine release and C-reactive protein concentrations following the administration of EPA for 1 month (Wigmore et al, 1997*c*). CRP may be used as a marker for pro-inflammatory cytokine activity in vivo. In the present cohort of patients, the proportion with an elevated CRP remained stable throughout the study period. In patients with advanced pancreatic cancer receiving no specific intervention, CRP tends to rise with disease progression (Falconer et al, 1994*a*). The provision of fish oil supplementation provided by the trial supplement in the current study may have prevented this progressive rise in CRP. A prospective randomized trial would be required to address this issue more formally.

Polyunsaturated fatty acids such as EPA have been shown to have an inhibitory effect on human pancreatic carcinoma cell lines in vitro (Falconer et al, 1994*b*). These effects may occur via cell cycle arrest and the induction of apoptosis (Lai et al, 1996). EPA will also slow the growth of experimental tumours in mice (Beck et al, 1991) and it is possible that part of the effect upon cachexia seen in the present study was secondary to inhibition of tumour growth. However, serial tumour imaging in pancreatic cancer is

© 1999 Cancer Research Campaign difficult to interpret and expensive and was thus not performed.

Overall survival of patients with pancreatic cancer is very poor with a median of 4.1 months (Ahlgren, 1996). Median survival from diagnosis in the present study was over 8 months although a condition of enrolment was that survival was expected to be over 2 months. Similar conditions apply to most chemotherapy trials where median survival of untreated patients has been noted to be between 63 and 122 days and overall median survival of treated patients to be between 160 and 170 days (Fearon et al, 1996). Clearly, overall survival in this study is at the upper end of that seen in chemotherapy trials but without the side-effects associated with chemotherapy. A recent randomized, controlled study of a mixed fish oil preparation (providing around 3 g of EPA and 2 g of DHA daily) in a group of patients with advanced cancer has suggested a modest survival benefit for those patients receiving fish oil (Gogos et al, 1998).

The present small pilot study suggests that a fish oil-enriched nutritional supplement has the potential to be a safe, effective anti-cachectic agent, in contrast to conventional nutritional supplements. A randomized, controlled trial would be required to confirm the observed anti-cachectic effect, evaluate any effect on survival and reveal any as yet undetected side-effects. Such a study is currently underway.

### ACKNOWLEDGEMENTS

The authors wish to thank Prof. DC Carter, Prof. OJ Garden, Mr S Paterson-Brown, Mr KK Madhavan, Mr GGP Browning, Mr JR Richards, Mr DAD MacLeod, Mr A Smith, Mr C Auld, Dr AJ MacGilchrist and Dr AJK Williams for allowing access to their patients during this trial. The authors would also like to thank Miss Rosemary Richardson and Mrs Alison Hinds for dietary analysis. This work has been supported by Ross Products Division, Abbott Laboratories and the Royal Infirmary of Edinburgh NHS Trust

### REFERENCES

Ahlgren JD (1996) Epidemiology and risk factors in pancreatic cancer. *Semin Oncol* **23**: 241–250

Barber MD, Wigmore SJ, Ross JA and Fearon KCH (1997) Eicosapentaenoic acid attenuates cachexia associated with advanced pancreatic cancer. *Prostaglandins Leukot Essent Fatty Acids* **57**: 204

Beck SA, Smith KL and Tisdale MJ (1991) Anticachectic and antitumour effect of eicosapentaenoic acid and its effect on protein turnover. *Cancer Res* **51**: 6089–6093

Cohn SH, Vartsky D, Vaswani AN, Sawitsky A, Rai K, Gartenhaus W, Yasumura S and Ellis KJ (1982) Changes in body composition of cancer patients following combined nutritional support. *Nutr Cancer* **4**: 107–119

DeWys WD, Begg C, Lavin PT, Band PR, Bennett JM, Bertino JR, Cohen MH, Douglass HO, Engstrom PF, Ezdinli EZ, Horton J, Johnson GJ, Moertel CG, Oken MM, Perlia C, Rosenbaum C, Silverstein MN, Skeel RT, Sponzo RW and Tormey DC (1980) Prognostic effect of weight loss prior to chemotherapy in cancer patients. *Am J Med* **69**: 491–497

Evans WK, Nixon DW, Daly JM, Ellenberg SS, Gardner L, Wolfe E, Shepherd FA, Feld R, Gralla R, Fine S, Kemeny N, Jeejeebhoy KN, Heymsfield S and Hoffman FA (1987) A randomised trial of oral nutritional support versus ad lib nutritional intake during chemotherapy for advanced colorectal and non-small-cell lung cancer. *J Clin Oncol* **5**: 113–124

Falconer JS, Fearon KCH, Plester CE, Ross JA and Carter DC (1994*a*) Cytokines, the acute-phase response, and resting energy expenditure in cachectic patients with pancreatic cancer. *Ann Surg* **219**: 325–331

Falconer JS, Ross JA, Fearon KCH, Hawkins RA, O'Riordain MG and Carter DC (1994*b*) Effect of eicosapentaenoic acid and other fatty acids on the growth in vitro of human pancreatic cancer cell lines. *Br J Cancer* **69**: 826–832

Falconer JS, Fearon KCH, Ross JA, Elton R, Wigmore SJ, Garden OJ and Carter DC (1995) Acute-phase protein response and survival duration of patients with pancreatic cancer. *Cancer* **75**: 2077–2082

Fearon KCH, Falconer JS, Ross JA, Carter DC, Hunter JO, Reynolds PD and Tuffnell Q (1996) An open-label phase I/II dose escalation study of the treatment of pancreatic cancer using lithium gammalinolenate. *Anticancer Res* **16**: 867–874

Gogos CA, Ginopoulos P, Salsa B, Apostolidou E, Zoumbos NC and Kalfarentzos F (1998) Dietary omega-3 polyunsaturated fatty acids plus vitamin E restore immunodeficiency and prolong survival for severely ill patients with generalised malignancy. A randomised controlled trial. *Cancer* **82**: 395–402

Hannan WJ, Cowan SJ, Plester CE, Fearon KCH and DeBeaux A (1995) Comparison of bio-impedence spectroscopy and multi-frequency bio-impedence analysis for the assessment of extracellular and total body water in surgical patients. *Clin Sci* **89**: 651–658

Heinrich PC, Castell JV and Andus T (1990) Interleukin-6 and the acute phase response. *Biochem J* **265**: 621–636

Jelliffe DB (1966) The assessment of the nutritional status of the community. WHO Monograph 53. WHO: Geneva

Jensen MB, Hermann AP, Hessov I and Mosekilde L (1997) Components of variance when assessing the reproducibility of body composition measurements using bio-impedence and the Hologic QDR-2000 DXA scanner. *Clin Nutr* **16**: 61–65

Lai PBS, Ross JA, Fearon KCH, Anderson JD and Carter DC (1996) Cell cycle arrest and induction of apoptosis in pancreatic cancer cells exposed to eicosapentaenoic acid in vitro. *Br J Cancer* **74**: 1375–1383

Leaf A and Weber PC (1988) Cardiovascular effects of n-3 fatty acids. *N Engl J Med* **318**: 549–557

Loprinzi CL, Schaid DJ, Dose AM, Burnham NL and Jensen MD (1993) Body-composition changes in patients who gain weight while receiving megestrol acetate. *J Clin Onc* **11**: 152–154

McMillan DC, O'Gorman P, Fearon KCH and McArdle CS (1997) A pilot study of megestrol acetate and ibuprofen in the treatment of cachexia in gastrointestinal cancer patients. *Br J Cancer* **76**: 788–790

McNamara MJ, Alexander HR and Norton JA (1992) Cytokines and their role in the pathophysiology of cancer cachexia. *JPEN J Parenter Enteral Nutr* **16** Suppl: 50S–55S

Meydani SN, Lichtenstein AH, Cornwall S, Meydani M, Goldin BR, Rasmussen H, Dinarello CA and Schaefer EJ (1993) Immunological effects of National Cholesterol Education Panel Step-2 diets with and without fish-derived n-3 fatty acid enrichment. *J Clin Invest* **92**: 105–113

Moldawer LL and Copeland EM (1997) Proinflammatory cytokines, nutritional support, and the cachexia syndrome. Interactions and therapeutic options. *Cancer* **79**: 1828–1839

Ovesen L, Hannibal J and Mortensen EL (1993*a*) The interrelationship of weight loss, dietary intake, and quality of life in ambulatory patients with cancer of the lung, breast, and ovary. *Nutr Cancer* **19**: 159–167

Ovesen L, Allingstrup L, Hannibal J and Mortensen EL, Hansen OP (1993*b*) Effect of dietary counseling on food intake, body weight, response rate, survival, and quality of life in cancer patients undergoing chemotherapy: a prospective, randomised study. *J Clin Oncol* **11**: 2043–2049

Perez MM, Newcomer AD, Moertel CG, Go VLW and DiMagno EP (1990) Assessment of weight loss, food intake, fat metabolism, malabsorption, and treatment of pancreatic insufficiency in pancreatic cancer. *Cancer* **52**: 346–352

Shizgal HM (1990) Validation of the measurement of body composition from whole body bioelectric impedence. *Infusionstherapie* **17** suppl **3**: 67–74

Simons JPFHA, Aaronson NK, Vansteenkiste JF, ten Velde GPM, Muller MJ, Drenth BM, Erdkamp FLG, Cobben EGM, Schoon EJ, Smeets JBE, Schouten HC, Demedts M, Hillen HFP, Blijham GH and Wouters EFM (1996) Effects of medroxyprogesterone acetate on appetite, weight, and quality of life in advanced-stage non-hormone-sensitive cancer: A placebo-controlled multicenter study. *J Clin Oncol* **14**: 1077–1084

Simons JPFHA, Schols AMWJ, Hoefnagels JMJ, Westerterp KR, JF, ten Velde GPM and Wouters EFM (1998) Effects of medroxyprogesterone acetate on food intake, body composition, and resting energy expenditure in patients with advanced nonhormone-sensitive cancer. *Cancer* **82**: 553–560

Tisdale MJ (1996) Inhibition of lipolysis and muscle protein degradation by EPA in cancer cachexia. *Nutrition* **12** Suppl: S31–33

Todorov P, Cariuk P, McDevitt T, Coles B, Fearon K and Tisdale M (1996) Characterisation of a cancer cachectic factor. *Nature* **379**: 739–742

Wigmore SJ, Ross JA, Falconer JS, Plester CE, Tisdale MJ, Carter DC and Fearon KCH (1996) The effect of polyunsaturated fatty acids on the progress of cachexia in patients with pancreatic cancer. *Nutrition* **12** Suppl: S27–S30

© 1999 Cancer Research Campaign 

Wigmore SJ, Plester CE, Richardson RA and Fearon KCH (1997*a*) Changes in nutritional status associated with unresectable pancreatic cancer. *Br J Cancer* **75**: 106–109
Wigmore SJ, Plester CE, Ross JA and Fearon KCH (1997*b*) Contribution of anorexia and hypermetabolism to weight loss in anicteric patients with pancreatic cancer. *Br J Surg* **84**: 196–197
Wigmore SJ, Fearon KCH, Maingay JP and Ross JA (1997*c*) Down-regulation of the acute-phase response in patients with pancreatic cancer cachexia receiving oral eicosapentaenoic acid is mediated via suppression of interleukin-6. *Clin Sci* **92**: 215–221

**Appendix 1** Composition of the trial fish oil-enriched nutritional supplement. Between-batch coefficients of variation in the proportion of EPA and DHA in the supplement were <1% and <1.5% respectively

| Nutrient | Amount per can (237 ml) |
|---|---|
| Energy (kcal) | 310 |
| Protein (g) | 16.1 (21% of calories) |
| Carbohydrate (g) | 49.7 (61% of calories) |
| Fat (g) | 6.5 (18% of calories) |
| EPA | 1.09 |
| DHA | 0.46 |
| Vitamin A (IU) | 1320 |
| Vitamin D (IU) | 192 |
| Vitamin E (IU) | 72 |
| Vitamin K (IU) | 32 |
| Vitamin C (mg) | 156 |
| Folic acid (µg) | 456 |
| Thiamin (mg) | 1.6 |
| Riboflavin (mg) | 1.2 |
| Vitamin B6 (mg) | 1.2 |
| Vitamin B12 (µg) | 4.32 |
| Niacin (mg) | 9.6 |
| Choline (mg) | 126 |
| Biotin (µg) | 187 |
| Pantothenic acid (mg) | 6 |
| Sodium (mg) | 360 |
| Potassium (mg) | 480 |
| Chloride (mg) | 365 |
| Calcium (mg) | 432 |
| Phosphorus (mg) | 300 |
| Magnesium (mg) | 108 |
| Iodine (µg) | 42 |
| Copper (mg) | 1.5 |
| Manganese (mg) | 0.6 |
| Zinc (mg) | 7 |
| Iron (mg) | 5.3 |
| Selenium (mg) | 22 |
| Chromium (µg) | 30 |
| Molybdenum (µg) | 49.4 |

 *© 1999 Cancer Research Campaign*
